# Evaluating the Impact of the “Gender Equity Movement in Schools” (GEMS) Intervention on Gender Knowledge and Attitudes Among Male Adolescents in Bangladesh: A Mixed‐Methods Cross‐Sectional Study

**DOI:** 10.1002/hsr2.71695

**Published:** 2025-12-29

**Authors:** Sudipta Das, Hridi Hedayet, Umme Haney, Nurjahan Akter, Sultana Sadia Islam, Md. Sanwarul Hoque Khan, Syed Shariful Islam, Fariha Haseen

**Affiliations:** ^1^ Department of Public Health and Informatics Bangabandhu Sheikh Mujib Medical University Shahbag Dhaka Dhaka Division Bangladesh; ^2^ Concerned Women for Family Development (CWFD) Banasree Dhaka Dhaka Division Bangladesh

**Keywords:** adolescent, attitude, Bangladesh, gender equity, gender role, gender‐based violence, Knowledge, male adolescents

## Abstract

**Background and Aims:**

The “Gender Equity Movement in Schools” (GEMS) intervention, a health education program for all adolescents in grades VI to VIII, intended to promote their knowledge of gender issues and transform their attitudes; however, the changes are found to be less significant among males. This study aimed to explore the gender issue‐related post‐intervention changes among male adolescents.

**Methods:**

This was a mixed‐methods study involving a survey of 234 male adolescents and two focus group discussions with 10 male adolescents from grades VII and VIII across two schools in Bangladesh (one of which received the GEMS intervention and the other did not). Quantitative data were collected using a self‐administered questionnaire incorporating the Gender Attitudinal Scale (GATS) and analyzed using SPSS‐25. Qualitative data were gathered through focus group discussions (FGDs) and analyzed using thematic analysis.

**Results:**

Knowledge regarding gender issues was found to be significantly higher among the GEMS‐exposed. Knowledge on gender equality issues was approximately three times higher (2.8, 95%CI = 1.404–5.714) in the GEMS exposed group compared to that of the non‐GEMS exposed group, while knowledge on gender issues amongst those who got the GEMS diary was approximately six times (5.9, 95%CI = 1.199–29.497) higher compared to that of those who did not receive the diaries. However, the total GATS score was high among the non‐intervention‐exposed adolescents, indicating better gender‐equitable attitudes. The quantitative and qualitative findings revealed that some parental and familial factors (higher parental education levels, maternal involvement in decision‐making and employment, and broader family participation) may influence the contradiction between increased knowledge and less favorable gender attitudes among the GEMS‐exposed male adolescents.

**Conclusion:**

The GEMS intervention increased male adolescents' knowledge; however, its effect on their gender attitudes appeared limited due to the influence of parental and familial factors, which may be vital for transforming these attitudes.

## Summary

- Among male adolescents, the Gender Equity Movement in Schools (GEMS) intervention has successfully improved knowledge regarding gender issues.
- As per our study findings, the GEMS intervention's impact on gender attitudes among male adolescents was found not to meet the expectations, highlighting a potential gap between knowledge acquisition and attitude transformation.
- Key factors, as per our study findings, that are possibly responsible for significant attitudinal changes among male adolescents included parental influences and family values. Future interventions targeting male adolescents to achieve more effective results regarding gender attitudinal changes should consider these factors.

## 1 Introduction

Achieving gender equality is critical for sustainable development globally. However, recent data reveal a concerning trend that the global community is not on the way to meet the United Nations' Sustainable Development Goal 5, which aims to achieve gender equality by 2030 [1, 2, 3]. While Bangladesh ranks first in the South Asian region and 59th globally in closing the gender gap, the country continues to fight with significant gender‐based challenges, including violence against women and the prevalence of child marriage [4]. Alarmingly, 72.6% of ever‐married women in Bangladesh experience some form of partner violence throughout their lives, and 50% of women aged 20–24 were married before the age of 18, with over 27% marrying before age 16 [5, 6]. These entrenched societal gender norms not only jeopardize the health and well‐being of women and girls but can also have detrimental effects on their families and communities [7].

The persistent unfavorable gender attitudes pose a significant challenge to achieving gender equality, particularly among adolescent boys and men, who are often socialized to support patriarchal values and resist challenging traditional norms [8, 9, 10, 11]. Studies show that many young men in Bangladesh conform to conventional gender norms that sustain inequalities. A national survey revealed that nearly two‐thirds of unmarried male adolescents believe sons should receive more education than daughters, and a significant proportion justifies the idea that women should adhere to their husbands' decisions [12]. This highlights the urgent need for interventions that foster positive gender attitudes among young people.

Early adolescence, specifically between the ages of 10 and 14, is a pivotal period for shaping gender attitudes and social norms [13, 14]. Interventions during this stage can significantly influence young people's perceptions and beliefs about gender, providing an opportunity to cultivate equitable attitudes [11, 15]. The Gender Equity Movement in Schools (GEMS) is one such initiative aimed at transforming gender attitudes, promoting equality, and preventing gender‐based violence by actively involving adolescents in discussions about gender issues [16].

This study aims to assess and compare the knowledge and attitudes toward gender and related issues among male adolescents exposed to the GEMS intervention and those who were not, focusing on gender concepts, discrimination in roles and attributes, child marriage, and violence against women. By identifying potential associated factors following the completion of the second phase of the GEMS intervention in selected schools, this research seeks to contribute valuable insights to the ongoing discourse on gender equity and the transformative potential of educational interventions in Bangladesh.

### 1.1 Overview of the GEMS Intervention and Literature Review

The GEMS intervention is a health education program designed to change adolescents' knowledge, attitudes, and practices regarding gender issues. The intervention provided a dissonance‐based strategy featuring four strategic pillars designed to motivate boys and girls to challenge norms, consider alternatives, and engage in gender‐transformative processes to address and prevent violence [16, 17]. Starting at a young age, involving boys and girls in gender discussion, adopting a gender‐transformative strategy, and utilizing institutional settings for normative change were the strategic pillars [16]. Initially developed and trialed in Mumbai, India, the GEMS intervention was launched in Vietnam (Da Nang), India (Jharkhand), and in Bangladesh (Barguna, Barishal, Dhaka, and Patuakhali districts) in 2012 under the auspices of UNFPA, with the Bangladesh Ministry of Education later adopting the project as the “Generation Breakthrough Project” [16, 17, 18]. The first phase of the GEMS initiative began in 2014, spanning 350 schools, and aimed to engage students in discussions on gender equity through various activities and sessions spread over two academic years [16, 17]. The core component of the intervention included gender equity activities (over two academic years, there were 26 sessions over nine modules to be discussed in the classroom as an extracurricular activity‐based program), GEMS school campaign (series of games, role‐playing exercises, competitions, and other activities to encourage discussion among all students), GEMS diary (a student book with different activities to reinforce the classroom sessions, teacher training and support, school orientation meetings, and parent and community outreach program [16, 17, 18]. The nine modules contained several sessions on gender and sex, gender discrimination, gender equity and equality, understanding violence, its effects, preventive measures and causes, impact, and prevention of child marriage [16, 17]. It also included lessons on adolescent sexual and reproductive health, understanding emotion and relationships, drug addiction and HIV/AIDS, and the communication process [16, 17]. The trained school teachers covered four modules in the first year and the remaining five in the second year.

After completing the first phase in 2016 in Bangladesh, the second phase was launched in July 2018 in 250 schools and madrasahs in the five districts (Jamalpur, Moulvibazar, Patuakhali, Rangamati, and Sirajganj) [16, 17, 18]. However, the second phase of the intervention faced challenges due to the COVID‐19 pandemic, which disrupted its implementation [18].

The intervention in Vietnam, India, and Bangladesh (Phase 1) was evaluated following its implementation; however, it yielded somewhat uneven results. The gender‐segregated data in India showed that, following the intervention, boys had shifted more significantly towards positive gender attitudes compared to girls. In contrast, in Vietnam and Bangladesh (end line), girls demonstrated a more positive attitudinal transformation than boys [16, 17]. Across all countries studied, those participants who attended more classes demonstrated significantly greater positive transformation [16, 17]. However, the findings were quite evident that boys tended to participate in those classes less frequently compared to girls.

In Bangladesh, the first phase was evaluated, but the second phase was not. This study′s intention was not to assess the intervention; instead, it aimed to observe & explore the effect on adolescent boys following its implementation in the second phase, which had previously been unexplored in Bangladesh. The primary objective of this study was to investigate the impact of the GEMS intervention on adolescent behaviors, with a focus on knowledge and attitudes toward gender issues.

## 2 Methods

From here on, the school with GEMS intervention will be termed a GEMS school, and the school without GEMS intervention will be referred to as a non‐GEMS school.

### 2.1 Study Design and Setting

This school‐based, comparative cross‐sectional study used a mixed‐methods research design. It was conducted in Sirajganj, one of the five districts selected for the second phase of the GEMS intervention program in Bangladesh.

For administrative purposes, Bangladesh is divided into eight divisions, which are further subdivided into 64 districts, and each district is divided into upazilas. Sirajganj District, located within the Rajshahi Division, comprises a total of 9 upazilas. Sirajganj Sadar Upazila was purposively selected from the district's nine upazilas through convenience sampling, chosen to ensure feasibility and accessibility and to expedite the data collection process. The proximity and homogeneity of the schools enabled an effective comparison between those participating in the GEMS program and non‐participating schools. To deepen the analysis, this study utilized Gender Schema Theory (GST) as a theoretical framework to explore how ingrained societal gender norms shape adolescents' health behaviors. According to GST, individuals internalize societal gender expectations early in life, forming cognitive schemas that influence their perceptions, interactions, and decision‐making [19]. This theoretical lens provides a robust structure for examining how adolescents process gendered information and how these cognitive schemas shape their engagement with health‐related behaviors and interventions. The application of GST in this context enables a nuanced understanding of the pathways through which gender norms are reinforced and acted upon in health behaviors among adolescents.

### 2.2 Participants, Sample Size, and Sampling Method

The GEMS sessions began with students in grade VI in January and concluded in March 2022. As a result, the grade VI students from the 2022 batch were not included in this study due to incomplete GEMS sessions. Therefore, the study included students from grades VII and VIII from both the GEMS school and the non‐GEMS school.

In the GEMS school, male students had varying levels of participation in the program, with some attending classroom‐based GEMS sessions, others receiving the GEMS diary, and some being involved in other components of the GEMS intervention. To ensure targeted sampling, male students who participated in the GEMS sessions and those who received the GEMS diary were purposively selected for this study. To fulfill the remaining sample size, additional students were conveniently recruited based on the study′s inclusion criteria (male students from grades VII and VIII of the selected schools, who volunteered to participate in this study) and their presence at the school during the data collection period.

In contrast, in the non‐GEMS school, male students from classes VII and VIII were selected using a systematic random sampling method, with the class roll list as the sampling frame. This ensured a structured and unbiased selection process for comparison with the GEMS school cohort.

#### 2.2.1 Quantitative Phase

The study participants were male students in classes VII and VIII from both the GEMS school and the non‐GEMS school, who were available during the data collection period and participated voluntarily. The sample size for the quantitative part was calculated using the following standard formula [20]:

$$n=\frac{{\left\{u\sqrt{\left[\pi (1-\pi )\right]}+v\sqrt{\left[\pi_{0}(1-\pi_{0})\right]}\right\}}^{2}}{{\left(\pi -\pi_{0}\right)}^{2}}$$

Here, *n =* required sample size; *u* = 1.96 for a 5% significance level; v (critical value for the power of the test) = 0.84; *π* = 60% (0.60), it is the approximate change in the percentage of adolescents who become gender sensitive following GEMS curriculum implementation. At the baseline, the rate was 38.8%; at the end‐line, the percentage increased to 98.6%, representing a 59.8% (approx. 60%) change [16, 17]; $\pi_{0}$= 50% (0.5), the expected level of change.

$$\text{So},\;n=\frac{{\left\{1.96\sqrt{\left[0.60\left(1-0.60\right)\right]}+0.84\sqrt{\left[0.50\left(1-0.50\right)\right]}\right\}}^{2}}{{\left(0.60-0.50\right)}^{2}}$$

$$n=\frac{{\left\{1.96\sqrt{0.24}+0.84\sqrt{0.25}\right\}}^{2}}{{\left(0.1\right)}^{2}}$$

$$n=\frac{{\left\{0.96+0.42\right\}}^{2}}{{\left(0.1\right)}^{2}}$$

$$n=\frac{1.9044}{0.01}$$

n=190.44≅191

So, the required sample size was approximately 191. The estimated sample size was 240 (after rounding), considering a 20% missing data rate due to the sensitive nature of the study topic, which was to be obtained from both schools. We calculated the adjusted sample size by using the formula: Adjusted sample size = *n*/(1 – dropout rate).

#### 2.2.2 Qualitative Phase

This study incorporated two focus group discussions (FGDs), one from the GEMS school and another from the non‐GEMS school, to gather qualitative insights from male adolescents about their gender attitudes, experiences, and perceptions of the GEMS program. Each FGD included five male participants from Class VIII, selected using purposive sampling to ensure that participants had relevant exposure to the program and could provide meaningful input. In total, 10 male students were involved in the FGDs.

### 2.3 Data Collection Tool

#### 2.3.1 Quantitative Part

A questionnaire was used to collect quantitative data. Variables were first identified in relation to the objectives when developing the data collection tool. Then, the questionnaire and appropriate measurement scale for each variable were determined based on the literature review and the baseline and end‐line evaluation study of the first phase of the Generation Breakthrough project. The Gender Attitudinal Scale (GATS), a validated scale with 20 statements, was previously used in baseline and end‐line Generation Breakthrough project evaluation surveys in Bangladesh and was employed in this study [16, 17]. All the statements were made unidirectional after the pre‐test and were internally consistent (Cronbach′s alpha = 0.819). The respondents' answers were categorized as strongly agree, agree, disagree, strongly disagree, and do not know. The attitudinal score for each respondent was calculated by summing the scores from their responses to 20 statements on gender issues. These statements were numbered, and the combined scores created an individual attitudinal score on the Gender Attitudes Scale, allowing for an overall assessment of each respondent′s stance on gender‐related topics.

#### 2.3.2 Qualitative Part

In the qualitative phase, a guideline was used for FGDs to explore their gender attitudes. The guideline was developed based on literature reviews and identified knowledge gaps through an interim analysis of the quantitative part after 50% of the data collection was completed.

The FGD guideline contained four themes: gender roles and responsibilities, gender attributes and attitudes, gender‐based violence, and experiences with the GEMS curriculum.

### 2.4 Data Collection Procedure

Following ethical approval from the Institutional Review Board of Bangabandhu Sheikh Mujib Medical University (BSMMU) and official permission from the District Education Office in Sirajganj, the data collection team commenced fieldwork in the selected schools. After receiving permission from the District Education Officer, we formally notified the respective head teachers of the selected schools to seek approval from them and to obtain verbal consent from the parents of the students in the selected grades. After getting the approval, data collection was initiated. The data collection team consisted of male medical doctors with a background in public health, selected specifically to facilitate rapport with the male adolescent participants, given the study′s focus on gender‐related issues, including gender concepts, gender discrimination, child marriage, and violence against women, in both GEMS and non‐GEMS schools.

Before data collection, written informed consent was obtained from the head teachers, followed by verbal consent from the classroom teachers. A separate room had been pre‐arranged for the data collection, ensuring privacy and comfort for the participants. Groups of 5–10 male students were escorted to the designated room, where they were seated individually. Written informed assent was then obtained from the students after explaining the study′s purpose, their role in it, and detailed instructions for completing the questionnaire. The pre‐tested, semi‐structured, self‐administered Bangla questionnaire was distributed, and participants were assured of confidentiality. Side conversations and copying were discouraged, and students were encouraged to ask questions if they found any item confusing. Upon completing the questionnaire, the data collection team reviewed the responses to ensure no omissions, thanked the participants, requested that they not discuss the questions with others, and then returned the participants to their classrooms. The entire process took approximately 50–60 min for each group. This protocol was uniformly followed in both the GEMS and non‐GEMS schools for quantitative data collection.

For the FGDs, participants were selected purposively from the same class and section to foster a comfortable and open discussion environment. Each FGD consisted of a moderator, a note‐taker, and the respondents, lasting approximately 50–60 min. The discussions were conducted in Bangla, the native language of both the participants and the research team. The moderator initiated the sessions with a few ice‐breaking questions to establish rapport, which helped participants feel at ease. The FGDs were audio‐recorded with the respondents' consent, and every effort was made to ensure privacy and confidentiality throughout the process. The data collection period extended from November to December 2022.

### 2.5 Data Analysis

#### 2.5.1 Quantitative Statistical Analysis

All the questionnaires were checked after the data collection was completed. Six data collection sheets were rejected due to incomplete, non‐understandable, and overwritten responses. According to the questionnaire, a data file template was developed in SPSS 25, and data regarding all questions were entered into the database. No outliers were found, and missing values were identified.

The quantitative statistical analysis used descriptive and inferential statistics, including student *t*‐test, chi‐square test, and linear and logistic regressions. Descriptive statistics, such as means and standard deviations, were calculated for age, other demographic variables, and GATS score. Proportions were used to summarize categorical variables, such as parental education, occupation, intervention exposure, respondents' knowledge, et cetera. The mean GATS score was compared using a Student′s t‐test (equal variances assumed). Chi‐square tests were used to assess the associations between categorical variables and respondents' knowledge and attitudes towards gender issues. Linear regression was employed to explore the relationship between respondents' GATS score and GEMS session attendance; Binary logistic regression was used to estimate odds ratios with 95% confidence intervals to explore the relationships between predictor variables, like GEMS session exposure, possessing GEMS diary, et cetera, and dependent variable, including knowledge of respondents regarding gender issues, after recoding those variables as dichotomous variables. In the binary logistic regression, we assessed the model fitness using the Hosmer‐Lemeshow test, where the *p*‐value > 0.05 indicated good model fit, and Nagelkerke *R* ^2^, and we estimated multicollinearity based on the tolerance ( > 0.2) and variance inflation factor ( < 5). All tests were two‐tailed, and statistical significance was set at the p‐value of less than or equal to 0.05 (*p* < 0.05).

#### 2.5.2 Qualitative Thematic Analysis

For qualitative data analysis, the process involved downloading recordings and transcribing them, with researchers adhering to field notes and recordings. Field notes were finalized on the day of the interview and cross‐referenced with the transcripts. A priori codes were developed based on Focus‐Group Discussion (FGD) guidelines, with additional inductive codes generated through repeated review of transcripts. A priori codes were further broken down into subcodes. Fifteen a priori codes, four themes, and eight sub‐themes were hence developed. Transcripts were organized according to predefined codebooks, facilitating data clustering, comparison, and theme identification. Thematic analysis was then manually conducted to explore emerging patterns and insights. The study adhered to the Consolidated Criteria for Reporting Qualitative Research (COREQ) checklist.

### 2.6 Ethical Considerations

Based on the Declaration of Helsinki and CIOMS International Ethical Guidelines, the Institutional Review Board (IRB) of Bangabandhu Sheikh Mujib Medical University (BSMMU) checked for the study′s adherence to the ethical guidelines and provided ethical clearance (Ref: BSMMU/2022/8752—Date: 31‐08‐2022). Official permissions were obtained from the United Nations Population Fund (UNFPA), Concerned Women for Family Development (CWFD), the District Education Officer, Sirajganj, and the head teachers of the selected schools. The ethical issues were strictly maintained throughout the research. Informed written consent was obtained from the head teachers, as the legally authorized representative, after getting verbal consent from parents, and informed written assent was obtained from the adolescents. The research details, including procedures for maintaining participants' privacy and confidentiality, obtaining informed consent from head teachers after getting their parental approval, and assent from students, with permission from their class teachers, were explained to the participants. Their privacy was protected, and their data will be kept confidential. The reporting standard was consistently ensured by employing the STROBE checklist from the CONSORT guidelines.

## 3 Results

### 3.1 Background Characteristics of Participants

A total of 234 male adolescents participated in the study, with a 100% response rate from the GEMS intervention school and a 94.9% response rate from the non‐GEMS school. Among these participants, approximately 51.7% were from the GEMS‐intervention school and 48.3% from the GEMS‐non‐intervention school, with a mean age of 14.05 years (± 1.095). Among the 121 students exposed to the GEMS intervention, 45 participants (37.2%) attended the sessions directly. Over the years, 13 sessions were held, with attendance rates varying: 51.1% attended four sessions or fewer, 35.6% attended five to ten sessions, and only 13.3% attended eleven or more sessions. Around 61% of GEMS students had diaries, but only 18.9% of them completed the diaries. Approximately 49% expressed satisfaction with the sessions, while 20% reported dissatisfaction (Tables 1 and 2).

**Table 1:** Socio‐demographic profile of the respondents (N = 234).

Variables	GEMS intervention status of the schools		GEMS intervention status of the schools	χ^2^ value	*p* value
	GEMS non‐intervention school *n* (%)	GEMS non‐intervention school *n* (%)			
Mother's education				43.626	**< 0.001**
No formal education or not known	30 (47.6%)	33 (52.4%)	63 (26.9%)		
Primary education	53 (86.9%)	8 (13.1%)	61 (26.1%)		
Secondary and above	38 (34.5%)	72 (53.1%)	110 (47.0%)		
Father's education a				46.100	**< 0.001**
No formal education or not known	37 (49.3%)	38 (50.7%)	75 (32.8%)		
Primary education	53 (86.9%)	8 (13.1%)	61 (26.6%)		
Secondary and above	29 (31.2%)	64 (68.8%)	93 (40.6%)		
Mother's occupation				0.578	0.447
Unpaid, care work	103 (50.7%)	100 (49.3%)	203 (86.8%)		
Paid work	18 (58.1%)	13 (41.9%)	31 (13.2%)		
Father's occupation b				74.536	**< 0.001**
Informal occupation	83 (74.8%)	28 (25.2%)	111 (49.1%)		
Formal occupation	33 (26.0%)	94 (74.0%)	127 (50.9%)		
Mother's involvement in family decision‐making				0.565	0.452
Mother involved	24 (47.1%)	27 (52.9%)	51 (21.8%)		
Mother not involved	97 (53.0%)	86 (47.0%)	183 (78.2%)		
Mother's involvement in activities other than household chores				3.845	0.050
Mother involved	24 (40.7%)	35 (59.3%)	59 (25.2%)		
Mother not involved	97 (55.4%)	78 (44.6%)	175 (74.8%)		

*Note:* Bold values statistical significance *p* < 0.05.

^a^ Missing value = 5

^b^ Missing value = 8

**Table 2:** GEMS (Gender Equity Movement in Schools)‐intervention profile of the respondents from the GEMS school (N = 121).

Variables		*n* (%)
Participation in GEMS sessions	Participant	45 (37.2%)
	Non‐participant	76 (62.8%)
Number of GEMS sessions attended	≤ 4 sessions	23 (51.1%)
	5–10 sessions	16 (35.6%)
	≥ 11 sessions	6 (13.3%)
Possession of GEMS diary	Possessed GEMS diary	74 (61.2%)
	Didn′t possess GEMS diary	47 (38.8%)
Completion of GEMS diary	Completed completion	14 (18.9%)
	Incomplete completion	40 (54.1%)
	Non‐completion	20 (27.0%)
Satisfaction after GEMS participation	Very satisfied	14 (31.1%)
	Satisfied	22 (48.9%)
	Disappointed	3 (6.7%)
	Very disappointed	6 (13.3%)

### 3.2 Knowledge About Gender Issues

The results in Table 3 demonstrate that the GEMS program successfully raised awareness among adolescents about various forms of GBV and the services available to address them. The stark contrast between the GEMS‐intervention group and the non‐intervention group in terms of knowledge about GBV illustrates the effectiveness of targeted education. Students in the GEMS‐intervention school demonstrated significantly higher levels of knowledge about gender issues, including the harmful effects of gender‐based violence (GBV) (*p* < 0.001) and awareness of violence‐related services (*p* < 0.001) (Table 3).

**Table 3:** Gender and GBV (Gender based violence)‐related knowledge profile of the respondents.

Variables	GEMS intervention status of the schools		Total n (%)	χ^2^ value	*p* value
	GEMS Intervention School n (%)	GEMS non‐intervention School n (%)			
Source of knowledge about gender issues (N = 139)				23.307	**< 0.001**
Family (parents and siblings)	4 (40.0%)	6 (60.0%)	10 (7.2%)		
School (teachers and friends)	66 (75.9%)	21 (24.1%)	87 (62.6%)		
Social media	14 (33.3%)	28 (66.7%)	42 (30.2%)		
Consideration of child marriage as GBV (N = 234)				9.792	**0.002**
Yes	77 (60.6%)	50 (39.4%)	127 (54.7%)		
No	42 (40.0%)	63 (60.0%)	105 (45.3%)		
Knowledge about negative effects of violence (N = 234)				17.305	**< 0.001**
Had knowledge	87 (63.0%)	51 (37.0%)	138 (59.0%)		
Didn′t have knowledge	34 (35.4%)	62 (64.6%)	96 (41%)		
Knowledge about violence‐related services (N = 234)				31.389	**< 0.001**
Had knowledge	86 (68.8%)	39 (31.2%)	125 (53.4%)		
Didn′t have knowledge	35 (32.1%)	74 (67.9%)	109 (46.6%)		
Knowledge about helpline number (N = 234)				8.487	**0.004**
Had knowledge	95 (57.9%)	69 (42.1%)	164 (70.1%)		
Didn′t have knowledge	26 (37.1%)	44 (62.9%)	70 (29.9%)		

*Note:* Bold values statistical significance *p* < 0.05.

Approximately 70% of GEMS students identified beating as a violent act, compared to only 42% of students in the non‐intervention school. This disparity indicates that, without intervention, many adolescents may fail to recognize certain harmful behaviors as forms of violence. The GEMS program appears to bridge this gap by providing students with the knowledge necessary to identify violence in its various forms, helping them to challenge and rethink behaviors that may be normalized in their communities. Furthermore, the finding that 68.1% of GEMS students recognized forced marriage as GBV highlights the program's success in broadening students' understanding of gender issues beyond physical violence. Forced marriage, often culturally ingrained in many societies, is a complex form of gender‐based violence that may not always be seen as such by younger individuals (Table 4).

**Table 4:** Distribution of respondents according to their knowledge about gender‐based violence (Violence Against Women) issues (N = 234).

Variables	GEMS school (N = 121)	Non‐GEMS school (N = 113)
	Frequency (percentage)	Frequency (percentage)
Activities involved in gender‐based violence (multiple responses)		
1. Physical violence		
Slapping	68 (57.6%)	37 (32.7)
Kicking	59 (50.0%)	43 (38.1%)
Punching	64 (54.2%)	43 (38.1%)
Hitting	80 (66.7%)	64 (56.6%)
Beating	85 (70.8%)	61 (54.0%)
Choking	67 (56.8%)	61 (54.0%)
Burning	62 (52.5%)	55 (48.7%)
Shoving	46 (39.0%)	23 (20.4%)
2. Psychological violence		
Threatening or stalking	57 (48.3%)	57(50.4%)
Throwing bad comments	63 (53.4%)	57 (50.4%)
3. Sexual violence		
Proposals to have physical contact	63 (53.4%)	70 (61.9%)
Sending mobile SMS with offensive words or videos	62 (52.5%)	71 (62.8%)
Touching body parts	82 (68.9%)	85 (75.2%)
Forced sexual intercourse or rape	105 (86.8%)	99 (87.6%)
4. Forced marriage	81 (68.1%)	65 (57.5%)
5. Child marriage	77 (64.7%)	50 (44.2%)
6. Wife beating	89 (74.2%)	73 (64.6%)
7. Acid throwing	85 (70.8%)	80 (70.8%)
Type of negative effects caused by GBV (Multiple responses)		
Physical Injury/Disability	61 (70.1%)	41 (80.4%)
Sexual and Reproductive health problems	57 (65.5%)	24 (47.1%)
Depression/Fear/Anxiety	50 (57.5%)	26 (51.0%)
Committing suicide or growing suicidal tendencies	64 (73.6%)	43 (84.3%)
Low self‐esteem/self‐confidence	50 (57.5%)	26 (51.0%)
Inability to attend school/madrasah	54 (62.1%)	26 (51.0%)
Avoiding social contact/activities	38 (43.7%)	16 (31.4%)
Parent/family members' humiliation in society	56 (64.4%)	33 (64.7%)
Financial costs of having health or legal services	66 (75.9%)	28 (54.9%)
Types of violence‐related services (multiple responses)		
One Stop Crisis Center	66 (76.7%)	27 (69.2%)
Public hospital	73 (84.9%)	27 (69.2%)
Private hospital/clinic	35 (40.7%)	18 (46.2%)
Counseling center under the Ministry of Women and Children Affairs (MOWCA)	55 (64.0%)	21 (53.8%)
Non‐Governmental Organizations (NGOs)	52 (60.5%)	18 (46.2%)
Victim support center in the police station	62 (72.1%)	32 (82.1%)
Shelter homes	27 (31.4%)	5 (12.8%)

The fact that non‐intervention students primarily emphasized the link between GBV and sexually violent activities highlights a narrower understanding of the issue, likely influenced by traditional gender narratives or a lack of comprehensive education. This underscores the need for tailored educational interventions like GEMS, which provide a broader, more nuanced view of gender‐based violence and its many forms, from physical abuse to forced marriages and emotional or psychological harm. The GEMS intervention not only enhanced students' knowledge of GBV but also significantly increased their awareness of violence‐related services. GEMS‐intervention students were three times more likely (OR = 2.832; CI = 1.404–5.714; *p* = 0.004) to know about services available for victims of violence than their non‐intervention counterparts (Table 5). This heightened awareness is crucial because simply recognizing violence is not enough; students need to be equipped with the tools and knowledge to seek help for themselves or others. The importance of knowing where to turn for support cannot be overstated, especially in contexts where victims may feel isolated or powerless to address their situations.

**Table 5:** Logistic regression predicting the likelihood of respondents' possession of knowledge about VAW‐related issues (N = 234).

Characteristics			Knowledge about the harmful effects of violence		Knowledge about violence‐related services		Knowledge about helpline number	
			OR (95% CI)	*p‐*value	OR (95% CI)	*p‐*value	OR (95% CI)	*p* value
Model 1 a	Exposure to GEMS intervention	Not exposed	Ref.					
		Exposed	1.501 (0.756– 2.981)	0.246	2.832 (1.404– 5.714)	**0.004**	1.525 (0.733– 3.177)	0.259
	Participation in GEMS sessions	Didn′t participate	Ref.					
		Participated	1.063 (0.305– 3.708)	0.923	0.744 (0.237– 2.342)	0.614	0.720 (0.195– 2.661)	0.623
	Number of GEMS sessions attended	≤ 5 sessions	Ref.					
		> 5 sessions	1.141 (0.197– 6.619)	0.883	5.202 (0.586– 46.195)	0.139	1.184 (0.204– 6.879)	0.850
	Possession of GEMS diary	No	Ref.					
		Yes	3.142 (0.881– 11.208)	0.078	5.946 (1.199– 29.497)	**0.029**	1.823 (0.490– 6.783)	0.370
	GEMS diary completion status	Not completed	Ref.					
		Completed (partially or fully)	1.205 (0.302– 4.812)	0.792	0.310 (0.060– 1.614)	0.164	1.606 (0.390– 6.619)	0.512
Model 2 b	Exposure to GEMS intervention	Not exposed	Ref.					
		Exposed	2.121 (0.878– 5.124)	0.095	3.728 (1.531– 9.077)	**0.004**	4.259 (1.516– 11.96)	**0.006**
	Participation in GEMS sessions	Didn′t participate	Ref.					
		Participated	0.941 (0.260– 3.403)	0.926	0.653 (0.195– 2.186)	0.489	0.515 (0.124– 2.143)	0.362
	Number of GEMS sessions attended	≤ 5 sessions	Ref.					
		> 5 sessions	1.303 (0.218– 7.783)	0.772	5.939 (0.653– 53.983)	0.114	1.329 (0.215– 8.212)	0.759
	Possession of GEMS diary	No	Ref.					
		Yes	3.314 (0.837– 11.727)	0.090	5.426 (1.056– 27.865)	**0.043**	1.786 (0.414– 7.704)	0.437
	GEMS diary completion status	Not completed	Ref.					
		Completed (partially or fully)	1.123 (0.270– 4.661)	0.873	0.313 (0.058– 1.677)	0.175	1.705 (0.368– 7.891)	0.495

*Note:* Bold values statistical significance *p* < 0.05.

^a^ Model 1: Crude analysis

^b^ Model 2: Adjusted for variables: Mother's education, Father's education, Mother's occupation, Father's occupation, Mother's involvement in family decision making, and other than household chores.

The study also found that having a GEMS diary—a tool used within the intervention to engage students in reflecting on gender issues—dramatically increased the likelihood of students knowing about these services. The nearly six‐fold increase in service awareness for those who completed their diaries (OR = 5.946; CI = 1.199–29.497; *p* = < 0.03) suggests that reflective practices play a key role in solidifying the knowledge imparted during the sessions (Table 5). The diary likely acted as a personal reinforcement mechanism, allowing students to process and internalize the information they received, making them more likely to remember and utilize the knowledge.

### 3.3 Attitudes Toward Gender Issues

**Figure 1:**
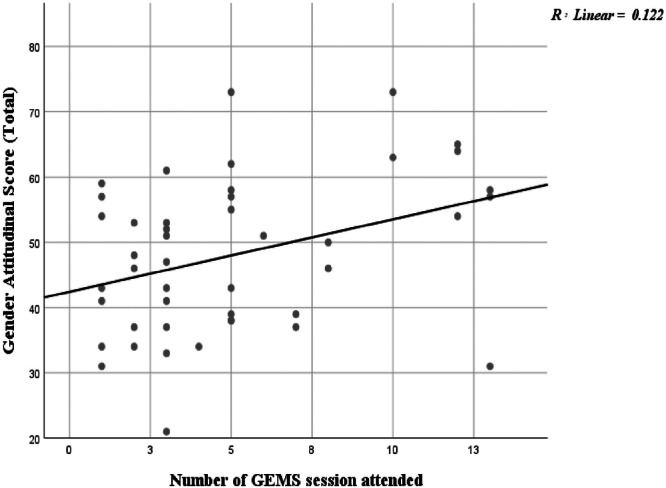
Relationship between the Gender Attitudinal Scale (GATS) score and the number of attended GEMS sessions. Scatter plot illustrating the positive linear relationship between male adolescents' GATS score (y‐axis) and the number of GEMS sessions attended by them (x‐axis) for 45 male adolescents from the GEMS‐exposed school, who participated in the GEMS sessions. The solid line represents the fitted linear regression model (y = 42.419 + 1.111x, *R* ^2^ = 0.122, *p* = 0.018). The regression analysis shows that each GEMS session participation increases the GATS score by 1.111 for male adolescents.

The comparison of gender attitudinal scores between the GEMS‐non‐intervention and GEMS‐intervention schools reveals an intriguing trend in the formation of gender attitudes among adolescents. The data show that students from the GEMS non‐intervention school had significantly higher gender attitudinal scores (Mean ± S.D. = 51.85 ± 11.742) compared to their counterparts in the GEMS intervention school (Mean ± S.D. = 47.02 ± 11.426), with a notable mean difference of −4.83 (*p* = 0.002). While this result appears paradoxical given the explicit focus of GEMS on promoting gender‐equitable norms, it underscores the complex and multifactorial nature of attitudinal formation. Several structural and contextual factors may account for this pattern. First, the non‐intervention schools may have benefited from pre‐existing sociocultural advantages, for instance, higher parental education, more gender‐equitable household practices, or school leadership that informally reinforces egalitarian norms even in the absence of a formal program. This interpretation is supported by the observed strong associations between positive gender attitudes and parental education, maternal employment, and maternal involvement in household decision‐making (Table 6). These findings suggest that the family environment has a substantial influence on gender norm internalization and can sometimes outweigh the effects of structured interventions. Second, the program implementation context may have influenced outcomes. GEMS schools are often selected in areas where traditional gender norms are more deeply entrenched, and thus, despite exposure to the intervention, baseline resistance to change might have constrained measurable attitudinal shifts. Third, response patterns and measurement sensitivity may have contributed to the apparent discrepancy. Students in non‐intervention schools, unexposed to critical dialog about gender, may have responded in a socially desirable manner, providing “ideal” answers aligned with perceived modern values. Conversely, GEMS participants who have engaged in reflective exercises and critical discussions may exhibit greater self‐awareness and more cautious responses, resulting in lower yet more authentic attitudinal scores. Despite the lower mean scores in the intervention group, the within‐group trend presents a more encouraging picture. A clear positive relationship was observed between the number of GEMS sessions attended and gender attitudes, as reflected in the Gender Attitudinal Scale (GATS) total score. Each additional GEMS session was associated with an incremental increase of 1.111 points on the GATS score, indicating a dose–response relationship between program exposure and attitudinal improvement (Figure 1).

**Table 6:** Association between respondents' attitude and their socio‐demographic characteristics (N = 234).

Variables		Attitude toward gender		χ^2^ value	*p* value
		Unfavorable *n* (%)	Favorable *n* (%)		
Mother's education	No education	16 (25.4%)	47 (74.6%)	9.750	**0.008**
	Primary education	23 (37.7%)	38 (62.3%)		
	Secondary and above	18 (16.4%)	92 (83.6%)		
Father's education	No education	21 (28.0%)	54 (72.0%)	8.242	**0.016**
	Primary education	21 (34.4%)	40 (65.6%)		
	Secondary and above	14 (15.1%)	79 (84.9%)		
Mother's occupation	Unpaid, care word	52 (25.6%)	151 (74.4%)	1.314	0.252
	Paid work	5 (16.1%)	26 (83.9%)		
Father's occupation	Informal occupation	26 (26.3%)	73 (73.7%)	0.776	0.378
	Formal occupation	27 (21.3%)	100 (78.7%)		
Mother's involvement in family decision‐making	Not involved	50 (27.3%)	133 (72.7%)	4.002	**0.045**
	Involved	7 (13.7%)	44 (86.3%)		
Mother's involvement in outside household chores	Not involved	49 (28.0%)	126 (72.0%)	4.994	**0.025**
	Involved	8 (13.6%)	51 (86.4%)		

*Note:* Bold values statistical significance *p* < 0.05.

### 3.4 Gender Role Perceptions

Gender Role Perceptions reflect how deeply embedded societal norms regarding gender roles influence young people′s views about the responsibilities of men and women. The traditional perspective expressed by some participants—

“*Men should work outside and earn for the family; women should take care of the house*”—illustrates the persistence of conventional gender schemas. This view is rooted in long‐standing cultural and social expectations that assign men the role of breadwinners and women the role of caregivers, reinforcing a division of labor based on gender. Such norms can perpetuate gender inequality, limiting women′s opportunities in the workforce and decision‐making spheres.

However, the GEMS (Gender Equity Movement in Schools) intervention appears to have created a space for rethinking these entrenched beliefs. Participants who underwent the GEMS program exhibited a shift in their perceptions, with one student remarking,

“After attending GEMS, I see that women can also work and should help in decision‐making at home.”

This indicates that the GEMS initiative was effective in challenging traditional gender schemas and promoting more egalitarian attitudes toward gender roles.

The transformative insights gained through GEMS reflect a significant departure from traditional gender roles, with students beginning to recognize women′s potential beyond domestic responsibilities. The intervention likely provided them with knowledge and examples that challenged their previously held views. By encouraging discussions around gender equality, the program facilitated critical thinking about social roles, highlighting that women can contribute to the workforce and participate in household decisions just like men.

This shift is important because it demonstrates the plasticity of gender schemas— the cognitive frameworks that individuals use to understand and perform gender. Gender schemas are typically learned early in life and become deeply ingrained, shaping perceptions of appropriate behaviors and roles for men and women. However, as shown by the GEMS program, these schemas can be reshaped through targeted education and exposure to new ideas. By challenging the default assumptions about gender roles, GEMS encourages students to reconsider traditional views and adopt more progressive attitudes, paving the way for future behavioral changes that promote gender equity.

The contrast between traditional and transformative views highlights the complexity of gender role perceptions in the lives of adolescents. While some participants continue to endorse traditional roles, others are open to changing their perspectives when exposed to new information. The GEMS program demonstrates that with consistent reinforcement through activities, discussions, and reflection, young people can develop a more nuanced understanding of gender roles that support equality and inclusion, ultimately contributing to a more gender‐balanced society.

### 3.5 Influences on Gender Attitudes

The conflicting messages within families and the broader social environment illustrate the complex factors that shape adolescents' gender schemas.

The comment, “*My father always said women are weak, but my mother works and manages everything too*,” reflects the contradictions young people face when reconciling traditional gender norms with the lived experiences of those around them.

This insight highlights how gender roles are not only transmitted through societal structures but also deeply influenced by the behaviors modeled within the family. These contradictory messages within the family setting reveal the cognitive dissonance adolescents may experience as they attempt to form coherent understandings of gender roles. Family members, particularly parents, serve as the primary socializing agents during early childhood, and their attitudes toward gender can have a profound impact on children′s beliefs. When these attitudes clash, it can create a space for critical reflection, prompting adolescents to question long‐held assumptions about the roles of men and women. The influence of both parents offers an essential dimension to understanding how traditional gender norms can be maintained or challenged within the home.

Peer influence also plays a critical role in reshaping gender attitudes.

The statement, “*When my friends talk about girls being equal to boys, it makes me think differently*,” underscores the power of social contexts, particularly during adolescence when individuals are highly susceptible to the opinions and behaviors of their peer group.

The significance of peer influence suggests that group‐based interventions, such as the GEMS program, which encourages open dialog about gender equity, can be highly effective in promoting attitude shifts. In addition to familial and peer influences, media representations were also identified as a key factor in altering perceptions of gender roles.

The participant′s observation, *“I see women in ads doing the same jobs as men, and it makes me realize they can do anything,”* highlights the increasing visibility of gender equality in media portrayals.

Media, particularly advertisements, television shows, and social media, play a pivotal role in shaping societal norms by either reinforcing traditional gender roles or presenting alternative representations. Positive depictions of women in roles traditionally dominated by men can serve as powerful counternarratives, challenging deeply entrenched beliefs about what men and women are capable of. In this case, the participant′s exposure to media content showing women performing tasks typically associated with men facilitated a shift in their understanding of gender roles.

## 4 Discussion

The findings from this study, conducted in a Bangladeshi context, align with global research on gender education interventions. Programs like the GEMS have been implemented in various parts of the world, often showing positive shifts in knowledge and attitudes toward gender‐based violence (GBV) and gender equality. Comparing and contrasting these findings with international research provides a nuanced understanding of how context, cultural factors, and intervention design influence outcomes.

A study in India evaluating the GEMS intervention found similar positive effects on adolescents' knowledge of gender issues. Specifically, students exposed to the intervention demonstrated significantly higher awareness of GBV and gender norms compared to non‐intervention groups [16, 21]. The increased awareness among Bangladeshi adolescents regarding forced marriage as a form of GBV mirrors findings from this Indian study, where harmful practices, including child marriage, were more readily identified as violence by intervention participants. However, the Indian GEMS intervention yielded slightly higher overall participation rates and completion of reflective tools such as diaries, suggesting cultural differences in how adolescents engage with structured educational programs. This might indicate that program engagement strategies need to be more tailored to the local context to improve participation and reflection in Bangladesh.

In contrast, a similar intervention in Uganda focused on addressing GBV in schools revealed mixed results. While students in the intervention group demonstrated improved knowledge about GBV, there was no significant difference in attitudes toward gender equality compared to the control group [22]. This is in stark contrast to the findings in Bangladesh, where participation in GEMS sessions was positively correlated with improved gender attitudes, indicating that repeated exposure to gender equality messages can gradually shape adolescent views. The relatively low gender attitudinal scores among intervention students in this study, despite their increased knowledge, also resonate with global findings that highlight the slow pace of attitudinal change, which may require more time and consistent exposure to fully internalize new gender norms [23].

The counterintuitive finding that non‐GEMS students' attitudinal scores were higher than those of GEMS participants needs careful consideration. Firstly, non‐GEMS schools might have implicit gender‐equitable practice built into them through modeling by teachers, shared leadership between men and women, or prior community involvement that influences student perceptions, regardless of formalized programming. Second, GEMS schools are generally in communities where patriarchal values are deep‐seated, creating a ceiling effect on measurable attitudinal change despite exposure. Third, students who are not exposed to direct gender curriculum may provide idealized answers, while GEMS participants, having been exposed to critical dialog, may be more reflective and thus less conformist to normative “gender‐correct” answers. Lastly, the psychometric sensitivity of the attitudinal scale can measure declarative endorsement of equality but perhaps not the depth or internalization of gender‐equitable reasoning, unwittingly rewarding superficial agreement over true attitudinal change.

The influence of family environment on gender attitudes, as shown in this study, has also been echoed globally. A study from the United States highlights that adolescents from households with egalitarian gender role models—where both parents share decision‐making and work responsibilities—demonstrate more progressive gender attitudes [24]. The positive association between maternal employment and gender attitudes in the current study supports this finding. Similarly, research from the Netherlands showed that children whose mothers were employed and participated in decision‐making were more likely to develop egalitarian views on gender [25]. These findings suggest that, regardless of geographical context, the family remains a crucial agent of socialization in shaping gender norms.

However, a critical difference between the current study and other global interventions is the degree to which peer influence and media shape adolescents' perceptions of gender roles. In studies from high‐income countries, such as one conducted in the UK, media and peer influence were found to significantly accelerate shifts in gender norms among adolescents [26]. The Bangladeshi participants' reflections on media portrayals of gender roles, such as women performing tasks traditionally associated with men, suggest that global media representations of gender equity are increasingly resonating in low‐income settings. The role of media as a change agent for gender norms might be underutilized in intervention design in Bangladesh, where targeted media campaigns could potentially reinforce the messages delivered through educational programs like GEMS.

While the study′s cross‐sectional nature, limited school coverage, and reliance on self‐reported data may affect generalizability, its mixed‐methods approach provides valuable insights and a foundation for more comprehensive future research.

In conclusion, while GEMS in Bangladesh has effectively enhanced adolescents' gender knowledge, the unexpectedly higher attitudinal scores among non‐GEMS students underscore the complex interplay of context, measurement sensitivity, and latent social influences in shaping gender attitudes. This finding highlights that attitudinal transformation is neither linear nor solely attributable to formal interventions. Furthermore, cultural and contextual adaptations, such as improving engagement with reflective tools and reinforcing media campaigns, could strengthen the long‐term attitudinal changes necessary to foster gender equality among adolescents.

## 5 Conclusion

The GEMS intervention effectively raised awareness and reshaped attitudes toward gender‐based violence and gender roles among adolescents, similar to gender education programs globally. However, the persistence of traditional gender attitudes in some participants underscores the need for ongoing, comprehensive interventions that address the family, peer, and societal influences on gender schemas. Comparisons with studies across different contexts reveal that while the GEMS program aligns with global trends in promoting gender equity, local cultural and familial factors play a crucial role in shaping the success of such interventions. Future efforts should focus on integrating gender education into broader societal changes that promote gender equality at all levels —from the household to the media —to achieve lasting change.

## Author Contributions

Conceptualization: Sudipta Das, Syed Shariful Islam, and Fariha Haseen. Methodology: Sudipta Das, Hridi Hedayet, Md. Sanwarul Hoque Khan, Syed Shariful Islam, and Fariha Haseen. Validation: Syed Shariful Islam and Fariha Haseen. Formal analysis: Sudipta Das, Hridi Hedayet, Syed Shariful Islam, and Fariha Haseen. Resources: Md. Sanwarul Hoque Khan. Data curation: Sudipta Das. Funding acquisition: Sudipta Das. Visualization: Sudipta Das, Hridi Hedayet, Umme Haney, Nurjahan Akter, and Sultana Sadia Islam. Supervision: Sudipta Das, Md. Sanwarul Hoque Khan, Syed Shariful Islam, and Fariha Haseen. Writing – Original draft: Sudipta Das, Hridi Hedayet, Umme Haney, Nurjahan Akter, and Sultana Sadia Islam. Writing – review and editing: Sudipta Das, Hridi Hedayet, Umme Haney, Nurjahan Akter, Sultana Sadia Islam, Md. Sanwarul Hoque Khan, Syed Shariful Islam, and Fariha Haseen.

## Disclosure

The lead author, Fariha Haseen, affirms that this manuscript is an honest, accurate, and transparent account of the study being reported; that no important aspects of the study have been omitted; and that any discrepancies from the study as planned (and, if relevant, registered) have been explained.

## Conflicts of Interest

The authors declare no conflicts of interest.

## Data Availability

The datasets used in this study are available from the corresponding author or lead author upon reasonable request.
